# A CTSA-based consultation service to advance research on special and underserved populations

**DOI:** 10.1017/cts.2020.6

**Published:** 2020-01-16

**Authors:** Nathalie Vizueta, Catherine A. Sarkisian, Peter G. Szilagyi

**Affiliations:** 1 Department of Pediatrics, UCLA Mattel Children’s Hospital, University of California, Los Angeles, Los Angeles, CA, USA; 2 Division of Geriatrics, David Geffen School of Medicine, University of California, Los Angeles, Los Angeles, CA, USA; 3 Veterans Affairs (VA) Greater Los Angeles Healthcare System Geriatric Research, Education, and Clinical Center, Los Angeles, CA, USA

**Keywords:** research consultation service, pediatrics, geriatrics, peer review, grant studio, grant review, faculty development, special populations, underrepresented minorities

## Abstract

In this report, we describe the implementation and short-term outcomes of a Special Populations Consultation Service within the University of California, Los Angeles (UCLA) Clinical and Translational Science Institute (CTSI). With the goal of increasing the quality and quantity of special population (SP) research, the UCLA CTSI Integrating Special Populations program designed a consultation service to support faculty and trainees conducting research involving one of three CTSI “special populations:” children, older adults, and/or minority; underserved; or health disparity populations. The Special Populations Consultation Service offers three types of activities: grant proposal studios, career consultations, and project reviews. UCLA CTSI faculty with appropriate content expertise serve as consultants. We evaluated this consultation model using satisfaction surveys and by quantifying funded grants and reported changes in career goals in SP research. Between 2016 and 2019, the Special Populations Consultation Service provided 59 consultations including 42 grant studios and was used by researchers at all levels from all four UCLA CTSI institutions. Recipients rated the consultations very highly. Funding success rates were 57% following K-level grant studios and 28% following R-level grant studios. Users of project and career consultations commonly attributed career accomplishments in part to their consultation experiences. The SP Consultation Service is feasible and acceptable and appears to enhance careers of investigators studying special populations.

## Introduction

The sociodemographic makeup of the United States is rapidly changing. In less than 15 years, non-Latino white persons will become the minority and adults over age 65 years will make up 25% of the American population [1]. Unfortunately, biomedical research lags far behind these demographic changes: the vast majority of subjects enrolled in National Institutes of Health (NIH) research studies are non-Latino whites [2,3], while children and adults aged over 75 years are routinely excluded from research studies [2,4,5].

As one step toward addressing the unmet need for more diverse research populations [2,6,7], in June 2016, we launched a new “Integrating Special Populations” (ISP) Research Core (https://www.ctsi.ucla.edu/about/programs/pages/specialpop) with support from the University of California, Los Angeles (UCLA) Chancellor’s office and a Clinical and Translational Science Award administered by the National Center for Advancing Translational Sciences (NCATS). The ISP core is within the UCLA Clinical and Translational Science Institute (CTSI) hub, with the overarching mission to improve the quality and quantity of science focused on three types of “special populations” as defined by NCATS [8]: children, older adults, and populations of persons impacted by health disparities (e.g., underrepresented minorities). Notably, NIH policy [7] now requires that children and older adults be included in all human subjects research conducted or supported by the NIH. Furthermore, there is tremendous need for high-quality scientific investigations asking and answering appropriate research questions in order to inform and address the wide (and in some cases growing) well-described pervasive health disparities based on race, ethnicity, and socioeconomic status. However, to our knowledge, there are no published evaluations of strategies focused on increasing the number of NIH investigators studying children, older adults, and minority; underserved; or health disparity populations in biomedical research at a Clinical and Translational Science Awards (CTSA) program hub.

Previous reports have described studios and/or consultation services as successful mechanisms to support community-based research [9–11]. “Grant studios” in particular have also been previously described as successful tools for improving proposal funding success rates, especially for junior faculty [12–17]. Building upon this previous body of research, our ISP leadership team created a “Special Populations Consultation Service” focused specifically on special population research. Because junior and mid-level faculty often struggle with grant writing, planning specific projects, and also career decisions[18,19], our goal was to provide expert consultations in these three dimensions of academic careers.

In this paper, we describe the implementation of the Special Populations Consultation Service and short-term outcome metrics. Our aims were to (1) assess the feasibility and acceptability of this consultation model and (2) estimate the impact based on self-reported metrics and grant funding outcomes.

## Methods

The Special Populations Consultation Service was designed for academic researchers conducting special populations (SP) research. Following NCATS, we defined SP research as research involving children, older adults, and/or populations affected by health disparities (e.g., racial/ethnic minorities and/or persons with lower socioeconomic status).

### Eligibility and Method of Solicitation

The Special Populations Consultation Service is available at no cost to all postdoctoral researchers and faculty members affiliated with any of the four institutions that comprise the UCLA CTSI: UCLA and its three partner institutions, Cedars-Sinai Medical Center, Lundquist Institute for Biomedical Innovation at Harbor-UCLA Medical Center, and Charles R. Drew University of Medicine and Science. We promoted the consultation service via CTSI flyers/online materials describing the service, word of mouth, and local presentations by ISP program staff and faculty.

### Consultation Service Team

The UCLA CTSI ISP core leadership team consists of two faculty co-directors funded at 20% effort, a full-time program manager and a part-time (50%) community outreach director. An executive committee including representatives from each of the three partner CTSI institutions meets regularly with the leadership team to provide substantive input on all ISP program activities, including the Special Populations Consultation Service. As the point person for consultation requests, the ISP program manager solicits appropriate expert consultants and reviewers, schedules consultations, and collects and distributes materials to/from consultants and clients. Potential consultants are selected from the pool of NIH-funded scientists across all UCLA CTSI institutions, based on appropriate content expertise as well as reputation for being skilled grant reviewers and/or project or career consultants. We additionally prioritized selecting consultants with research expertise (e.g., track record of NIH funding and/or peer-reviewed publication) in the NCATS CTSI-defined “special population” that was the focus of the consultation (e.g., pediatrics, geriatrics, and/or disparity population).

Faculty reviewers participated in the consultations without compensation.

### Consultations Services

The Special Populations Consultation Service provides three consultation types: (1) internal, presubmission grant reviews (here-after called “grant studios”), (2) career consultations, and (3) project-specific consultations. Consultations are generally conducted in-person with all expert consultants/reviewers together at one time along with the consultation service recipient; occasionally, reviewers call in by phone. Clients are encouraged to audiotape the consultation with consent from all consultants. Interested faculty/trainees doing SP research seeking a consultation submit an electronic “Consultation Service Intake Form” (see Supplementary material 1) and CV to the ISP program manager; the form is tailored to the specific type of consultation.

#### Grant proposal studios

Grant studios involve 2–3 senior faculty reviewers, plus an in-person grant studio moderator (usually one of the two ISP faculty co-directors). When a career development grant is being reviewed, the primary mentor sometimes attends as well. The studios are held 4–6 weeks prior to the targeted submission deadline. Potential grant mechanisms include NIH funding mechanisms (e.g., either career development awards (CDA) or any type of R-level application) and Patient-Centered Outcomes Research Institute (PCORI) proposals. The intake request form solicits details about the grant application (e.g., Funding Opportunity Announcement, funding agency, grant mechanism, target submission date, project title, and grant proposal keywords). The investigator is also asked to suggest potential reviewers and provide a date when a draft of the grant will be available. However, the ISP program reviews the work of prospective grant reviewers very carefully to select experienced, grant-funded faculty reviewers.

The ISP program manager then invites faculty members via electronic mail to serve as grant studio reviewers. The invitation includes a summary of the grant proposal details, as well as the applicant’s specific aims page (when available). After the grant studio has been scheduled, the applicant submits a grant draft proposal to the program manager via email 7 days prior to the scheduled grant studio. The program manager sends this to the reviewers and moderator along with an NIH-style review template and instructions. Reviewers complete and return their written reviews to the program manager and the moderator at least 1 day before the grant studio, allowing the moderator time to read the reviews; reviewers are also encouraged to send critiques directly to the applicant before the grant studio meets. Reviewers utilize an NIH-style nine-point rating scale (1 = exceptional; 9 = poor) to provide an overall impact score as well as individual criterion scores [20,21]. Reviewers provide brief written comments including strengths/weaknesses for each of the review criteria [22] (i.e., for R series: Significance, Investigator(s), Innovation, Approach, and Environment) and for CDA: Candidate, Career Development Plan/Career Goals & Objectives, Research Plan, Mentor(s), Co-Mentor(s), Consultant(s), Collaborator(s); and Environment & Institutional Commitment to the Candidate). Reviewers also make a recommendation to either submit the proposal as planned or to delay submission.

The grant studio lasts 60 minutes. The first half simulates a regular NIH study section except that the grant applicant is in the room as a silent observer. The moderator starts by asking each reviewer for his/her overall impact score, then the primary reviewer provides a brief overview of the proposal and a 5–10 minute detailed summary of his/her review. Then the next reviewer gives his/her review, focusing on areas where he/she disagrees with the first reviewer and/or identifies new issues. Reviewers respond to each other’s comments, and the moderator summarizes key points and differences of opinion.

During the second half of the hour, the client him/herself responds to the reviews, and the moderator and the reviewers work together to provide specific constructive feedback to help the grant applicant improve the application. In the final 5 minutes, the moderator and reviewers give their overall recommendations as to whether to submit as planned, or delay in order to allow time for recommended revisions; if substantial revisions are recommended, this recommendation will naturally depend upon how much time the client has to devote to revision before the submission deadline.

After the grant studio, the moderator typically spends another 20–30 minutes individually with the client to help collate and prioritize the critiques and modifications. In addition, reviewers frequently offer to be available to assist in the future with either a specific issue on the same proposal or a different proposal. If they have not done so before the grant studio, reviewers send their reviews directly to the applicant after the studio.

#### Career consultations

Suggested topics for a career consultation include (a) potential sources of funding, (b) collaboration opportunities, (c) the overall direction of the research, (d) promotion and advancement, (e) career transitions, and (f) strategies for achieving academic success. Career consultations are especially encouraged for faculty at career crossroads or transitions, e.g., near the end of a funding cycle, or deciding between different employment opportunities. Just as with grant studio clients, interested faculty/trainees seeking a career consultation submit an electronic “Consultation Service Intake Form” (see Supplementary material 1) and CV. In this case, the client is asked to briefly describe his/her career goals in the next 5 years and indicate the specific question(s) for which he/she seeks consultation. The client also indicates how many hours per week he/she can commit to research in the next year and delineates a projected plan regarding grant proposal and manuscript submissions, and/or research presentations at conferences (if applicable).

The ISP project manager then recruits and assembles an interdisciplinary team of 2–3 senior faculty consultants with appropriate expertise to provide one-time career advice and sends the collected information to the consultants and the moderator. The career consultation lasts 1 hour. First, the client describes his/her question. Next each consultant spends approximately 10 minutes giving specific feedback to answer the question, and then the moderator leads an interactive discussion in which the consultants and the client strategize together on specific next steps to address the question. Occasionally, one of the consultants will offer to connect the client with a potential mentor/collaborator or him/herself may offer to become a longer-term mentor and the relationship will extend beyond the one-time career consultation.

#### Project-specific consultations

Suggested topics for project-specific consultations include (a) conceptualizing a project, (b) identifying key collaborators, (c) identifying potential grant sources, (d) study design, (e) manuscript development, or (f) obtaining advice on operation-related issues such as IRB submissions, hiring staff, and subject recruitment and enrollment of special populations. The process for arranging and executing project-specific consultations is similar to that of career consultations, except with a focus on a specific project.

### Evaluation

After the consultation, the ISP program manager sends each consultation service recipient a link to an electronic post-consultation meeting survey via REDCap [23]. The survey adapted previously tested surveys developed by the UCLA CTSI Workforce Development program and the UCLA Children’s Discovery Institute. The post-consultation survey (see Supplementary material 2) is tailored using automatic branch patterns to the type of consultation and additionally includes general items measuring level of satisfaction with the quality of the consultation service, and whether or not the client would recommend the consultation service to their colleagues or department. The survey also probes the client’s opinion regarding the best feature of the consultation service, the extent to which the consultation service resulted in new opportunities, and how the client would improve the consultation service.

For grant studio consultations, the client indicates the extent to which the grant review session helped him/her prepare a stronger grant proposal, how much it changed and/or helped their NIH application, and whether he/she changed the timing of their grant submission based upon the grant studio. For career consultations, the client indicates the degree to which the career consultation changed and/or helped their career planning, whether or not they feel more confident about meeting their career goals, and whether or not they have a concrete plan following the consultation. For project-specific consultations, the client indicates the degree to which the consultation changed and/or helped his/her project and to specify whether and/or how he/she changed the project as a result of the consultation.

## Results

### Characteristics of Consultation Service Recipients

Between September 2016 and December 2019, the Special Populations Consultation Service provided 59 consultations (Table 1). The majority (58%) of clients were referred by their own department. Investigators from seven departments including nursing, pediatrics, public health, medicine, and psychiatry have used the service. Grant studios were the most common type of consultation (*n* = 42, 71%), followed by project specific (*n* = 13, 22%) and career consultations (*n* = 4, 7%). Overall, 64% of the consultations involved research focused on children; 16% involved research focused on older adults. Altogether, 51% focused on racial/ethnic minorities or other disparity population (some involved children, others older adults, and some involved all ages). Clients were most frequently female (69.5%); 12% self-identified as Latino and 12% as Black or African-American. Most were at the rank of Assistant Professor (64%), had NIH early stage investigator status (71%), and had an MD degree (51%). The Special Populations Consultation Service was utilized by investigators from all partner institutions.

**Table 1.** Characteristics of recipients and consultations between September 2016 and December 2019

Characteristic	*N* (%)
Source of referral	
Own department	34 (57.6%)
ISP leadership	7 (11.9%)
Other CTSI program^a^	8 (13.6%)
CTSI brochure or advertisement	7 (11.9%)
Center outside own department/CTSI^b^	3 (5.1%)
Gender	
Male	16 (30.5%)
Female	41 (69.5%)
Highest academic degree	
MD	30 (50.8%)
MD/PhD	11 (18.6%)
MD/DrPH	1 (1.7%)
PhD	13 (22%)
ScD	2 (3.4%)
MPH	1 (1.7%)
MA	1 (1.7%)
Academic rank or title	
Trainee (e.g., postdoctoral scholar or fellow)	3 (5.1%)
Clinical Instructor	3 (5.1%)
Assistant Professor	38 (64.4%)
Associate Professor	12 (20.3%)
Professor	2 (3.4%)
Other (e.g., Research Project Manager)	1 (1.7%)
Institutional affiliation	
University of California, Los Angeles	44 (75%)
Cedars-Sinai Medical Center	2 (3%)
Lundquist Institute/Harbor-UCLA Medical Center	10 (17%)
Charles R. Drew University of Medicine and Science	3 (5%)
Department	
Pediatrics	32 (54%)
Medicine	9 (15%)
School of Nursing	7 (12%)
School of Public Health	6 (10.9%)
Psychiatry and Human Behavior	2 (3%)
Pediatrics and Emergency Medicine	1 (2%)
Emergency Medicine	1 (2%)
Surgery	1 (2%)
Race^c^	
White	18 (30.5%)
Hispanic or Latino	7 (11.9%)
Black or African-American	7 (11.9%)
Asian	11 (18.6%)
Prefer not to answer	4 (6.8%)
Consultation type	
Grant Studio	42 (71%)
R01	12 (29%)
R21	6 (14%)
R03	2 (5%)
R34	1 (2%)
K08	4 (9%)
K01	5 (12%)
K23	10 (24%)
K24	2 (5%)
Project-specific Consultation	13 (22%)
Career Consultation	4 (7%)
Population focus of grant/career/project	
Children	25 (42%)
Children and group impacted by health disparity	13 (22%)
Older adults	4 (7%)
Older adults and group impacted by health disparity	5 (9%)
Group impacted by health disparity (not children or older adults)	12 (20%)

DrPH, Doctor of Public Health; ScD, Doctor of Science; MPH, Master of Public Health.

a Workforce Development, Administrative Core (Grants Submission Unit).

b UCLA Resource Centers for Minority Aging Research (RCMAR); Clinical Research Education and Career Development (CRECD) at CDU.

c Race/ethnicity data missing for 12 recipients.

### Post-Consultation Survey

**Fig. 1.**
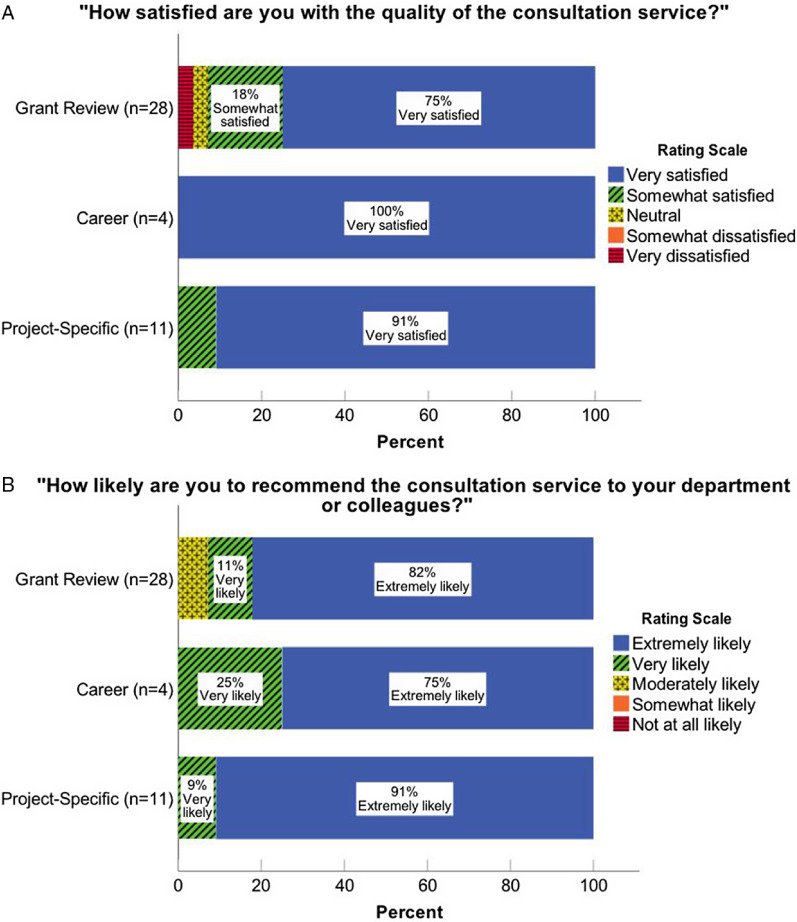
Ratings by consultation type indicating the (A) level of overall satisfaction with the quality of the consultation service and (B) likelihood to recommend the consultation service.

**Fig. 2.**
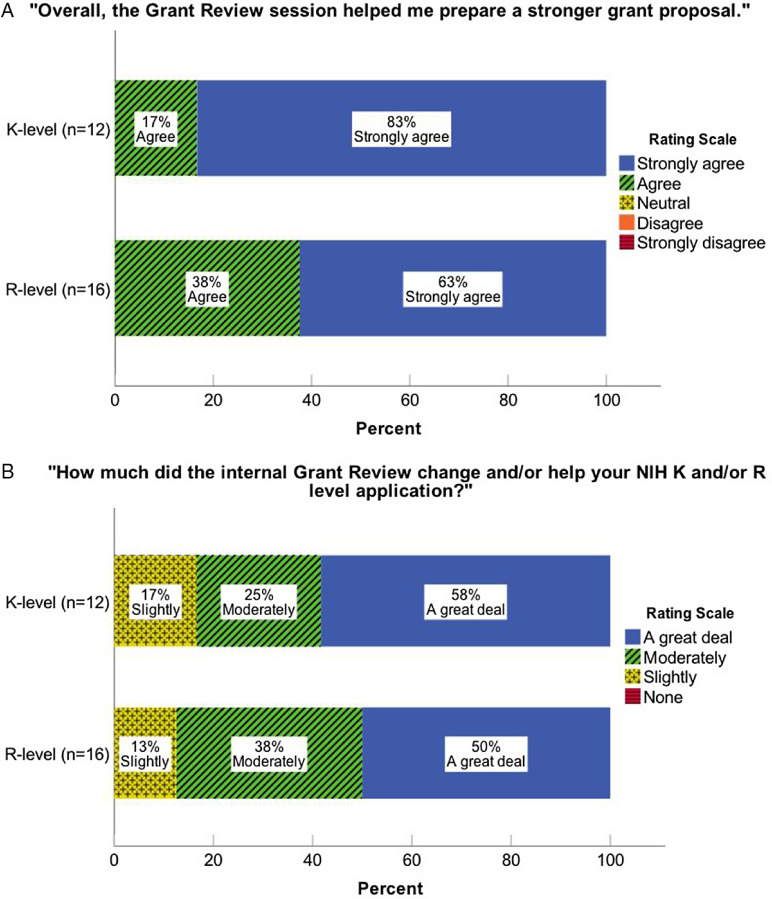
Ratings by grant type indicating the extent to which the grant studio (A) helped the investigator prepare a stronger proposal and (B) changed and/or helped the NIH application.

We began using the standardized Consultation Service Intake Request Form and the Post-Consultation Survey in January 2017. Of 49 clients sent the post-consultation survey, 88% (*n* = 43) completed and returned it. Figures 1 and 2 display results from the post-consultation survey. Across all consultation types, 81% of faculty reported that they were “very satisfied” with the quality of the consultation service (Fig. 1A). Further, 95% of faculty reported that they were either “extremely likely” or “very likely” to recommend the consultation to their department or colleagues (Fig. 1B). Of note (not shown), 25 (58%) consultation recipients^1^ reported that the consultation resulted in one or more new opportunities. Of these recipients, 15 reported that the consultation service resulted in a potential opportunity for a new collaboration, 14 reported that the consultation resulted in new awareness of literature, and 7 reported learning of a different grant funding mechanism/opportunity as a result of the consultation. Three investigators learned of a new career opportunity as a result of the consultation and two indicated they gained awareness of other CTSI services (albeit the latter was not a preset response option and may, therefore, be an underestimate).

### Grant Studio Outcomes (*n* = 42)

As shown in Table 1, 42 proposals (21 K-level and 21 R-level) were reviewed; of the R-level applicants specifically, 72% were from early stage investigators. R01 and K23 applications were the most frequently reviewed funding mechanism. Of note (not shown), 27 NIH applications were new submissions and an additional 7 were resubmissions^2^. Of these resubmissions, five were R grants and 2 were K grants; however, four of the R-level grants were sent to us for consultation as a resubmission because of poor score the first time (i.e., we did not review the application prior to the initial submission). All grant studio recipients reported that the consultation helped them prepare a stronger proposal (Fig. 2); most (86%) reported that the consultation changed or helped their application either “moderately” or “a great deal”. Not shown in the figure: 77% of grant studio recipients^3^ submitted their proposal on the date originally planned, with 23% delaying (and two applicants changing the funding mechanism without substantial delay.)

Four of the 42 grant applications received two mock study sections (i.e., prior to initial submission and prior to resubmission). The unit of analysis for funding outcomes is, therefore, 38 representing the number of unique grant applications. Grant submissions were stratified by award status (awarded, unfunded, pending). The success rate [12,14] was determined as: 100 × [(awarded)/(awarded+unfunded)]. Table 2 presents award status and success rates by grant type. Eighteen studio participants submitted 18 K-level applications with a success rate of 57% (8 awards/14 decisions; 4 pending). Fourteen studio participants submitted 17 R-level applications with a success rate of 28% (4 awards/14 decisions; 3 pending). To date, reviewed ISP grant studio applications have resulted in 12 awards and over 8.8 million^4^ dollars in funding (numbers reflect direct plus indirect dollars) including two R01s, one R03, one R21, two K08s, five K23s, and one K24.

**Table 2.** ISP grant studio success rates by grant type

Grant type	Success rate, *N* (%)^a^	Awarded	Unfunded	Pending NIH review^b^	Not submitted
CDA: K01, K08, K23, K24	8 of 14 (57%)	8	6	4	1
R01, R03, R21, R34	4 of 14 (28%)	4	10	3	2
Average, combined	12 of 28 (43%)	12	16	7	3

CDA, Career Development Award; ISP, Integrating Special Populations.

a ISP grant studio success rate (%) was determined as: 100 × [(awarded)/(awarded+unfunded)].

b Number of additional applications currently under NIH review provided for reference.

#### Career consultations (*n* = 4)

All clients who participated in a career consultation completed the evaluation and reported that the consultation helped either “moderately” (*n* = 2, 50%) or “slightly” (*n* = 2, 50%) with their career planning; all 4 reported feeling more confident about meeting their career goals and endorsed having a concrete career plan following the consultation.

#### Project-specific consultations (*n* = 13)

Of the clients who completed the evaluation (*n* = 11), 91% reported that the consultation changed/helped their project either “a great deal” (*n* = 6, 54%) or “moderately” (*n* = 4, 36%).

#### Consultant experience

To date, 58 of the 59 of the consultations included the participation of at least one senior faculty consultant with expertise in the CTSI “special population” (e.g. pediatrics, geriatrics, and/or disparity population) that was the focus of the consultation. Overall, 67 different consultants from five institutions and 18 departments, including leaders from 8 programs^5^ of the UCLA CTSI, have served as grant reviewers or senior consultants. Of the consultants, 70% (*n* = 47) had clinical/research expertise in the CTSI special population focus. Approximately two-thirds of invited reviewers accepted the invitation; the most common reason cited for declining the invitation was unavailability due to travel or personal grant proposal deadlines. For the grant studios, most consultants reported spending approximately 2–3 hours on their grant review. Though we did not formally measure faculty experience, most reported informally that they found participating in the Special Populations Consultation Service to be a positive experience, as evidenced by the fact that 90% (*n* = 19) of reviewers and senior consultants who were invited a second time agreed to participate.

## Discussion

We successfully implemented a new Special Populations Consultation Service designed for academic investigators at the UCLA CTSI hub. This consultation model has demonstrated feasibility and shows preliminary evidence of impact based upon client surveys indicating high satisfaction and reported change in career plans. Though the observational nature of this study precludes attributing causality, it is encouraging that the Special Populations Consultation Service was associated with relatively high rates of successful grant funding, particularly for career development grants.

Among the three types of consultations offered, the grant studios were the most frequently utilized. While the Special Populations Consultation Service was used predominately by women, junior faculty, investigators with MD degrees, and those conducting pediatrics research, the consultation service reached a wide range of clients, including mid-career and senior investigators, those with PhD or MD/PhD degrees, and those conducting disparities research. In addition, although the Special Populations Consultation Service targeted research focused on SP research and not under-represented minority (URM) researchers, it is interesting that 14 (24%) recipients were URM, a much higher proportion than exists among all researchers at UCLA. Overall, the Special Populations Consultation Service appeared to meet a need at our CTSI hub – namely, assistance with peer review of extramural funding applications for faculty applicants across multiple departments, regardless of academic rank.

Compared to formal grant studios described in the literature which were designed to reach prospective clients in the School of Nursing [13] or Departments of Neurology [12] at single institutions, the grant studios coordinated through the Special Populations Consultation Service were open to investigators across multiple departments at any of our four CTSI-affiliated institutions. While this wide scope presents challenges for finding reviewers with the appropriate research and/or methodological expertise, clients reported benefitting from receiving feedback from reviewers outside their department or institution. This is consistent with the recommendation by others [13] to seek faculty members in other schools or departments to serve as internal reviewers to help balance the quality of reviews; junior faculty, in particular, may not readily have access to senior faculty outside their department.

Preliminary outcomes for CDA, in particular, appeared to benefit from the grant proposal studios. Compared to the NIH CDA success rate of 32.5% in fiscal year 2018, we observed a high success rate, with half of CDA applications reviewed through the Special Populations Consultation Service being funded; this success rate is comparable to CDA funding outcomes reported for other grant studio recipients [12]. The R studios were not as successful based upon funding outcomes, although our sample size was small and many grant proposals are still being reviewed; longer follow-up is needed.

Our number of project-specific and career-specific consultations were small, and arguably underutilized. Nevertheless, frequently, clients reported that the consultation “resulted in a major change,” suggesting that measurable outcomes such as reduced burnout, successful grant funding, and promotions might be important downstream outcomes. For example, through the Special Populations Consultation Service, a junior faculty member in the Department of Pediatrics with a research background in statistical modeling was introduced to an implementation science framework that she was previously unaware of and introduced to an expert in homelessness research; she subsequently rewrote a CDA application and a new R21 application using this new framework and is developing a third grant application with the new collaborator.

There are a number of limitations to this small observational study. First, we did not formally assess the impact on the client for enhanced inclusion of special populations (SP) in the post-consultation survey. Second, as institutional comparators (e.g., a matched comparison group or baselines prior to the initiation of ISP grant studios) are not available, it is not possible to attribute precisely the degree to which grant studios helped grant applications or contributed to funding. Likewise, it is not possible to directly quantitate the degree to which the Special Populations Consultation Service resulted in more faculty or trainees conducting research or pursuing careers in SP. In the early days of this model, we have disproportionately drawn consultations from UCLA-based researchers and clients conducting pediatric research. This may be in part because grant review services are already accessible to geriatric researchers through the NIA-funded UCLA Resource Center for Minority Aging Research (RCMAR) in the Department of Medicine and the UCLA Pediatrics Department policy of requiring internal peer review for faculty who submit a NIH CDA or their first R01 grant.

### Future Directions

We are broadening our outreach to increase utilization of the Special Populations Consultation Service from investigators from other departments and the other three UCLA CTSI-affiliated institutions. Although the consultation service is moderately busy, there is capacity for growth across our four institutions. To date we have not had difficulty recruiting faculty reviewers and/or consultants, but as we expand, we are considering implementing an incentive system [24] with public accolades and/or small awards for outstanding reviews and/or consultations. We plan to suggest that reviewers add this service to their dossiers as examples of service to the institution, and we are considering paying reviewers. As applicant burden represents 75% of the system burden compared to reviewer and administrative burden, efforts are also needed to increase the value applicants receive by applying or reducing the level of burden [25]. Future evaluations will measure success rates of grants focused on new content areas and/or new methodologies, applicant burden, rates of burnout/career dropout, impact on retention rates, or intention to stay in SP research as well as promotion to leadership positions. A recent review [26] of programs focused on leadership for nursing researchers found that they have a positive influence on research productivity, including increase in publications and grant writing, improved leadership skills, and positively influenced health and well-being, staff relationships, work culture, and collaborations. Lastly, while we have reviewed only NIH grants so far, we have recently expanded the Special Populations Consultation Service to provide peer review for other large grant proposals to federal organizations or national foundations other than PCORI (e.g., Robert Wood Johnson).

## Conclusions

Implementing a Special Populations Consultation Service across multiple departments and institutions was feasible. Though future research is needed to examine long-term outcomes and expanded metrics, the Special Populations Consultation Service has become an important mechanism for increasing research focused on SP within the UCLA CTSI.

## Footnotes

1 Self-reported data regarding whether the consultation service resulted in new opportunities is missing, and/or survey was not available for administration to *n* = 16 clients.

2 Tracking data regarding whether grant was new or resubmission at time of grant review is missing for *n* = 8 clients.

3 Self-reported data regarding whether the timing of the grant submission changed due to the internal grant review is missing for *n* = 11 clients.

4 Total awarded amount is missing for 1 R01 and 1 K23 and is thereby an underestimate.

5 Workforce Development; Informatics; Network Resources; Community Engagement and Research Design; Participant and Clinical Interactions; Biostatistics, Epidemiology and Research Design; Precision Health; and Integrating Special Populations.

## Supplementary material

For supplementary material accompanying this paper visit https://doi.org/10.1017/cts.2020.6. click here to view supplementary material
