# Supplementary material for: A CTSA-based consultation service to advance research on special and underserved populations
Source: J Clin Transl Sci. 2020 Jan 16;4(4):271–8. doi: 10.1017/cts.2020.6 (PMC7681147; doi:10.1017/cts.2020.6)
Supplement: Supplementary file 1 [file S2059866120000060sup.zip › S2059866120000060sup001.pdf]

Consultation Service Intake Form

Please complete this form to help us coordinate your consultation.

What type of referral is this?
◯ Self-referral
◯ Referral for someone else

Triggers automated branching logic

Referral made by (Your last name, first name, and email) ______________________________

How did you hear about our consultation services?
☐ CTSI brochure or advertisement
☐ Own division or department
☐ Other (please specify)
(check all that apply)

____
(Please Specify (i.e. Name of person))

Please fill out the contact information below for the person you are referring:

First Name: ______________________________

Last Name: ______________________________

Email ______________________________

Degree(s): ______________________________

Department and Division (e.g. Medicine-Cardiology; School of Public Health-Epidemiology; Pediatrics-General Pediatrics) ______________________________

Institution:
◯ Cedars-Sinai
◯ Charles R. Drew University (CDU)
◯ UCLA-Westwood
◯ Harbor (LA BioMed)

Academic Title:
◯ Postdoc or Fellow
◯ Clinical Instructor (if not trainee)
◯ Assistant Professor
◯ Associate Professor
◯ Professor
◯ Other

____
(Please Specify)

The questions below are presented once to survey respondent (using REDCap's automated branching) depending on referral type selected above.

What type of consultation are you requesting? (Check One) Triggers automated branching logic

To learn more about the 3 consultation services we offer, please visit: https://www.ctsi.ucla.edu/researcher-resources/pages/specialpop

- ◯ Internal Pre-submission Grant Review
- ◯ Career Consultation
- ◯ Project-Specific Consultation
- ◯ I am not sure which consultation is most appropriate and I would like to speak with someone from your program.

What type of consultation are you recommending for Dr. [last_name]? Triggers automated branching logic

To learn more about the 3 consultation services we offer, please visit: https://www.ctsi.ucla.edu/researcher-resources/pages/specialpop

- ◯ Internal Pre-submission Grant Review
- ◯ Career Consultation
- ◯ Project-Specific Consultation
- ◯ I am not sure which consultation is most appropriate.

For FAQs regarding our grant studios (including eligibility), please visit: https://www.ctsi.ucla.edu/pages/specialpop_faqs

---

For FAQs regarding our grant studios (including eligibility), please visit: https://www.ctsi.ucla.edu/pages/specialpop_faqs

---

Which special population does your research project involve? (Check all that apply)

- ☐ Children
- ☐ Older Adults
- ☐ Population affected by health disparities (please specify)

(check all that apply)

Which special population does your grant involve? (Check all that apply)

- ☐ Children
- ☐ Older Adults
- ☐ Population affected by health disparities (please specify)

(check all that apply)

Population with disparities:

____
(Please Specify)

Briefly describe your health specialty/research focus:

____

Which special population does Dr. [last_name]'s research project involve (please check all that apply):

- ☐ Children
- ☐ Older Adults
- ☐ Population affected by health disparities (please specify)

(check all that apply)

Which special population does Dr. [last_name]'s grant involve (please check all that apply):

- ☐ Children
- ☐ Older Adults
- ☐ Population affected by health disparities (please specify)

(check all that apply)

Population with disparities:

____
(Please Specify)

Briefly describe Dr. [last_name]'s health specialty/research focus: ______________________

Briefly tell us why you are recommending Dr. [last_name] as a potential client: ______________________

The questions below are displayed based on the type of consultation selected above.

Grant Studio Questions

Please provide details of your grant application:

Please provide details of Dr. [last_name]'s grant application:

Funding Opportunity Announcement (FOA) ______________________
(i.e. what is online link to the FOA)

Funding Opportunity Announcement (FOA) ______________________
(i.e. what is online link to the FOA)

Check One
- ◯ New Submission
- ◯ Resubmission (if resubmission, what was the previous score and percentile?)

Check One:
- ◯ New Submission
- ◯ Resubmisssion (if resubmission, what was the previous score and percentile?)

Previous score and percentile: ______________________

Previous score and percentile: ______________________

Funding Agency (e.g. NIA, NIDDK, NIMH) ______________________

Funding Agency (e.g. NIA, NIDDK, NIMH) ______________________

Grant Mechanism (e.g. K08, K23, R21, R01) ______________________

Grant Mechanism (e.g. K08, K23, R21, R01): ______________________

Target Submission Date: ______________________

Target Submission Date: ______________________

Mentor(s) and/or Collaborator(s): __________________________________

(Please tell us who is on the grant)

Mentor(s) and/or Collaborator(s): __________________________________

(Please tell us who is on the grant)

Project Title: __________________________________

Project Title: __________________________________

Grant Proposal Keywords __________________________________

Grant Proposal Keywords __________________________________

Have you discussed this grant with a NIH program officer?

- ◯ Yes
- ◯ No

Please suggest potential reviewers: __________________________________

(do not include mentors/collaborators/consultants on grant)

Approximate date when you may have a draft of Specific Aims and Research Strategy to share with reviewers __________________________________

(NOTE: if K grant, the Training Plan would also need to be submitted for review)

Please answer the following 4 questions about your grant application:

Please answer the following 4 questions about grant application:

Does the study involve human subjects?

- ◯ Yes
- ◯ No
- ◯ I don't know

Does the study involve human subjects?

- ◯ Yes
- ◯ No
- ◯ I don't know

Are the participants prospectively assigned to an intervention?

- ◯ Yes
- ◯ No
- ◯ I don't know

Are the participants prospectively assigned to an intervention?

- ◯ Yes
- ◯ No
- ◯ I don't know

Is the study designed to evaluate the effect of the intervention on the participants?	◯ Yes<br>◯ No<br>◯ I don't know
Is the study designed to evaluate the effect of the intervention on the participants?	◯ Yes<br>◯ No<br>◯ I don't know
Is the effect that will be evaluated a health-related biomedical or behavioral outcome?	◯ Yes<br>◯ No<br>◯ I don't know
Is the effect that will be evaluated a health-related biomedical or behavioral outcome?	◯ Yes<br>◯ No<br>◯ I don't know
OPTIONAL: If you have any questions about our grant studios or would like to share additional information that would be helpful in coordinating your grant studio, please include here:	______________________________
OPTIONAL: If you have any questions about our grant studios or would like to share additional information that would be helpful in coordinating Dr. [last_name]'s grant studio, please include here:	______________________________

Career Consultation Questions

How many hours per week can you commit to research in the next year?	________________________
Briefly, what is your projected plan in the next year regarding grant submissions, manuscripts in preparation, and/or data presentations at conferences (if applicable)?	______________________________
Briefly, describe your career goals in the next 5 years?	______________________________
What specific questions do you need consultation about?	______________________________
Please suggest potential faculty consultant(s) who we should consider asking to participate in the meeting:	________________________
What specific questions does Dr. [last_name] need consultation about?	______________________________
Please suggest potential faculty consultant(s) who we should consider asking to participate in the meeting:	________________________

Project Specific Consultation Questions

What specific questions do you need consultation about? __________________________________

Please suggest potential faculty consultant(s) who we should consider asking to participate in the meeting: ___________________________

What specific questions does Dr. [last_name] need consultation about? __________________________________

Please suggest potential faculty consultant(s) who we should consider asking to participate in the meeting: ___________________________

CV and Specific Aims

Can we contact Dr. [last_name]?
- ◯ Yes
- ◯ No

REQUIRED: Please upload your CV here:

If available, please upload your Specific Aims here:

If available, please upload Dr. [last_name]'s CV here:

If available, please upload Dr. [last_name]'s Specific Aims here:

You indicated this is a resubmission. If available, please upload the NIH summary statement here: (NIH summary statement)
