# Supplementary material for: A CTSA-based consultation service to advance research on special and underserved populations
Source: J Clin Transl Sci. 2020 Jan 16;4(4):271–8. doi: 10.1017/cts.2020.6 (PMC7681147; doi:10.1017/cts.2020.6)
Supplement: Supplementary file 1 [file S2059866120000060sup.zip › S2059866120000060sup002.pdf]

Post-Consultation Survey

INSTRUCTIONS: Please evaluate the consultation service provided by the CTSI Special Populations Program. These questions help our program monitor usage of our consultation services and may help us improve program services. Your responses will be kept confidential and only be shared with the CTSI in aggregated form.

Contact Information

Please review the following information that you provided prior to your consultation. Does this information need to be updated or corrected in our records?

First Name: [first_name]
Last Name: [last_name]
Degree(s): [degree]
Email: [email]
Institution: [institution]
Department: [division]
Academic Title: [title]
Date of Consultation Service: [det_date]

◯ Yes
◯ No

Please update the fields that need change:

First Name: ______________________________

Last Name: ______________________________

Degree(s): ______________________________

Email: ______________________________

Institution

◯ Cedars-Sinai
◯ Charles R. Drew University
◯ UCLA-Westwood
◯ Harbor (LA BioMed)

Department/Division (e.g. Medicine/Cardiology) ______________________________

Academic Title:

◯ Postdoc or Fellow
◯ Clinical Instructor (if not trainee)
◯ Assistant Professor
◯ Associate Professor
◯ Professor
◯ Other

____
(Please Specify)

Date of Consultation Service: __________________________________

Evaluation

1. What type of Consultation Service did you participate in? Triggers automated branching logic

- ◯ Internal Pre-Submission Grant Review
- ◯ Career Consultation
- ◯ Project-Specific Consultation

(check one)

2. Please classify the RESEARCH discussed during your consultation:

- ☐ Basic Science is directed toward greater knowledge or understanding of the fundamental aspects of phenomena and of observable facts without specific applications towards processes or products in mind. Examples of basic research research in psychology might include: An investigation looking at what whether stress levels influence how often students engage in academic cheating. A study looking at how caffeine consumption impacts the brain.
- ☐ Clinical Research is research in which people, or data or samples of tissue from people, are studied to understand health and disease. Clinical research helps find a new and better ways to detect, diagnose, treat, and prevent disease. These studies also may show which medical approaches work best for certain illnesses or groups of people. Examples of clinical research include clinical trials, which test new treatments fro a disease, and natural history studies, which collect health information to understand how a disease develops and progresses over time.
- ☐ Health Services Research also known as health systems research or health policy and systems research examines how people get access to health care practitioners and health care services, how much care costs, and what happens to patients as a results of this care. The main goals of health services research are to identify the most effective way to organize, manage, finance, and deliver high quality care: reduce medical errors; and improve patient safety. HRS is more concerned with delivery and access to care, in contrast to clinical research, which focuses on the development and evaluation of clinical treatments.
- ☐ Other:

(check all that apply)

____
(Please Specify)

3. How satisfied are you with the quality of the consultation service?	◯ Very Satisfied<br>◯ Somewhat Satisfied<br>◯ Neutral<br>◯ Somewhat dissatisfied<br>◯ Very dissatisfied
4. How likely are you to recommend the consultation services to your department or colleagues?	◯ Extremely likely<br>◯ Very likely<br>◯ Moderate likely<br>◯ Somewhat likely<br>◯ Not at all likely
5. Did the consultation service result in new opportunities for you? (Please check all that apply)	☐ New awareness of literature<br>☐ Potential opportunity for new collaboration(s)<br>☐ Different grant funding<br>☐ New Career opportunity<br>☐ Other:
	______________________________<br>(Please Specify Other)
	______________________________<br>(Please tell us about the potential collaboration)
6. What was the BEST feature of the consultation service?	_____________________________________
7. How would you improve the consultation service?	_____________________________________
8. Please provide any other comments about your consultation service:	_____________________________________

The questions below are displayed based on the type of consultation that client is evaluating.

Grant Studio Questions

9. Overall, the Grant Review session helped me prepare a stronger grant proposal.	◯ Strongly Agree<br>◯ Agree<br>◯ Neutral<br>◯ Disagree<br>◯ Strongly Disagree
10. How much did the internal Grant Review change and/or help your NIH K and/or R level application?	◯ A great deal<br>◯ Moderately<br>◯ Slightly<br>◯ None
Grant Project Title:	______________________________

Grant Funding Agency (e.g. NIA, NIDDK): ______________________

Grant Mechanism (e.g. K08, K23, R21, R01): ______________________

11. Did you change the timing of your grant submission due to the internal grant review?

Target Submission Date before grant studio: [due_date]

- ◯ Submit on date orginally planned
- ◯ Delay
- ◯ Will no longer submit grant
- ◯ Other (please specify)

____

Date of Actual Grant Submission: ______________________
(Date you already submitted or plan to submit (following grant studio))

Career Consultation Questions

9. How much has the career consultation session changed and/or helped your career planning?

- ◯ A great deal
- ◯ Moderately
- ◯ Slightly
- ◯ None

10. Do you feel more confident about meeting your career goals?

- ◯ Yes
- ◯ No

11. Are you leaving the career consultation service meeting with a concrete plan?

- ◯ Yes
- ◯ No

Project Specific Consultation Questions

9. How much has the project-specific consultation session changed and/or helped your project?

- ◯ A great deal
- ◯ Moderately
- ◯ Slightly
- ◯ None

10. What was the most helpful part of the consultation? ______________________

11. Will you change anything as a result of the consultation (please specify)? ______________________

Demographic Information

Why am I being asked to provide gender, ethnicity and other information about myself? We are collecting demographic information in order to report on characteristics of participants who utilize our consultation services. This information will only be shared with the CTSI in aggregate form. Your personal Identity and identifying information will not be shared with unauthorized people. Please note that one of the response options provided is "prefer not to answer".

Gender
- ◯ Male
- ◯ Female
- ◯ Prefer not to answer

Do you consider yourself Hispanic/Latino?
- ◯ Yes
- ◯ No
- ◯ Don't Know
- ◯ Prefer not to answer

Please select the group that best represents your Hispanic origin or ancestry:
- ☐ Puerto Rican
- ☐ Dominican (Republic)
- ☐ Mexican
- ☐ Mexican American
- ☐ Chicano
- ☐ Cuban
- ☐ Cuban American
- ☐ Central or South
- ☐ Other Latin American
- ☐ Other Hispanic
- ☐ Don't Know
- ☐ Prefer not to answer

Other Latin American: ________________________________

Other Hispanic: ________________________________

Race:
- ☐ White
- ☐ Black/African American
- ☐ Native American
- ☐ Alaska Native
- ☐ Native Hawaiian
- ☐ Guamanian
- ☐ Samoan
- ☐ Other Pacific Islander
- ☐ Asian Indian
- ☐ Chinese
- ☐ Filipino
- ☐ Japanese
- ☐ Korean
- ☐ Vietnamese
- ☐ Other Asian
- ☐ Other
- ☐ Don't Know
- ☐ Prefer not to answer

Other Pacific Islander (please specify): ________________________________

Other Asian (please specify): ________________________________

____

Other Demographics (check all that apply):

- ☐ Person with disability
- ☐ Person from disadvantaged background (see definition below)
- ☐ Don't Know
- ☐ Prefer not to answer
- ☐ Not Applicable

Federal definition of disadvantaged background:
"Individuals who come from a family with an annual income below established low-income thresholds. These thresholds are based on family size; published by the U.S. Bureau of the Census; adjusted annually for changes in the Consumer Price Index; and adjusted by the Secretary for use in all health professions programs. The Secretary periodically publishes these income levels at http://aspe.hhs.gov/poverty/index.shtml. For individuals from low income backgrounds, the institution must be able to demonstrate that such participants have qualified for Federal disadvantaged assistance or they have received any of the following student loans: Health Professions Student Loans (HPSL), Loans for Disadvantaged Student Program, or they have received scholarships from the U.S. Department of Health and Human Services under the Scholarship for Individuals with Exceptional Financial Need."

And/or

"Individuals who come from a social, cultural, or educational environment such as that found in certain rural or inner-city environments that have demonstrably and recently directly inhibited the individual from obtaining the knowledge, skills, and abilities necessary to develop and participate in a research career."
